# Acute portal vein thrombosis secondary to COVID-19: a case report

**DOI:** 10.1186/s12876-020-01518-2

**Published:** 2020-11-19

**Authors:** Roham Borazjani, Seyed Reza Seraj, Mohammad Javad Fallahi, Zhila Rahmanian

**Affiliations:** 1 Student Research Committee, Shiraz University of Medical Sciences, Shiraz, Iran; 2 Department of Internal Medicine, Shiraz University of Medical Sciences, Shiraz, Iran; 3 Thoracic and Vascular Surgery Research Center, Shiraz University of Medical Sciences, Shiraz, Iran; 4 Department of Internal Medicine, Jahrom University of Medical Sciences, Jahrom, Iran

**Keywords:** COVID-19, Pneumonia, Portal vein, Thrombosis

## Abstract

**Background:**

COVID-19 pneumonia exhibits several extra-pulmonary complications.

**Case presentation:**

A 23-year old, asthmatic male with coronavirus pneumonia developed with generalized, acute abdominal pain. Further evaluations revealed a mild ascites and portal vein thrombosis although the patient received proper anticoagulation therapy. Routine lab data regarding the secondary causes of portal vein thrombosis were normal.

**Conclusion:**

We speculated that the underlying cause of portal vein thrombosis in our case was coronaviruses. Therefore, clinicians should always consider thrombosis and other hypercoagulable diseases in patients with COVID-19.

## Background

Novel Coronavirus pneumonia was first described as pneumonia of unknown cause in Wuhan, China, at the end of 2019 [1] and rapidly became pandemic. With time, some new extra-pulmonary manifestations of this viral pneumonia were described. Increased incidence of thromboembolic events was frequently reported [2].

Herein, we aim to describe a 26-year-old male with COVID-19 pneumonia and acute Portal Vein Thrombosis (PVT).

## Case presentation

A 26-year-old male, a known case of asthma, was brought to the Emergency Department (ED) in Faghihi hospital, Shiraz, Iran, on 11 April 2020 due to acute-onset dyspnea and a decrease in the level of consciousness since the day of admission. He was admitted with the impression of an acute asthma attack. There was no history of fever, hemoptysis, diarrhea, nausea, vomiting (N/V), lower gastrointestinal tract bleeding, incontinence, and stroke signs and symptoms. He had asthma for several years and only used the salbutamol inhaler as needed. His social history was positive for alcohol, cigarette smoking, and occasionally marijuana.

**Fig. 1:**
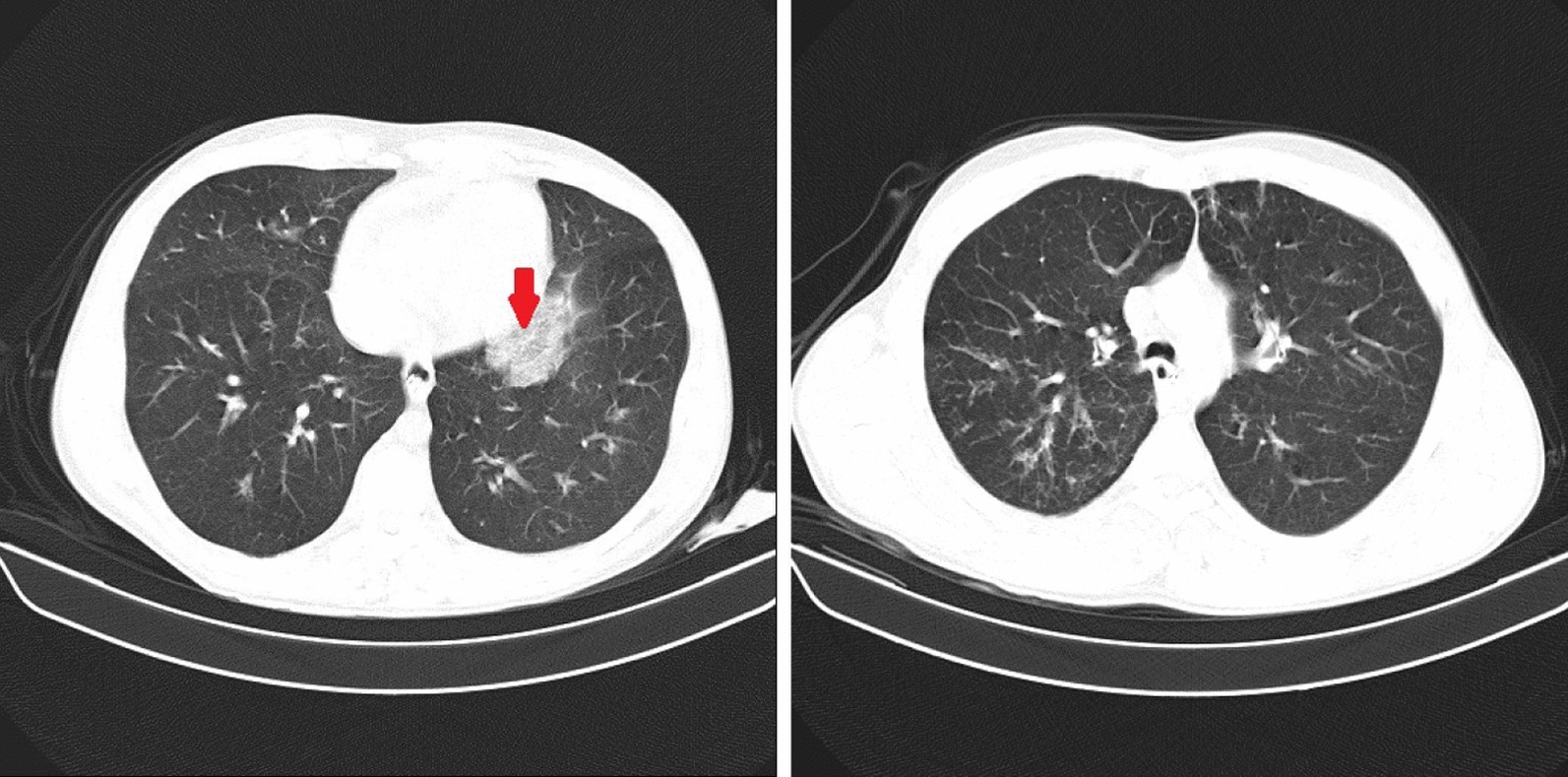
Chest CT showed bilateral peripheral and peribronchovascular patchy ground glass opacities (arrow) in both lungs with tree-in-bud appearance

We intubated the patient due to severe hypoxia and respiratory distress; blood Oxygen saturation (O_2_ Sat.) was 60% and 97% before and after intubation, respectively. The initial vital signs included temperature: 36.9 °C, pulse rate: 110/min, respiratory rate: 36/min, and blood pressure: 120/90 mmHg. Generalized wheezing was heard through auscultation. Other physical findings were normal. Brain computed tomography (CT) was normal. Chest CT showed bilateral peripheral and peribronchovascular patchy ground-glass opacities in both lung fields with tree-in-bud appearance (Fig. 1).The tracheal aspirate was positive for real-time polymerase chain reaction (RT-PCR) for SARS-COV2. The blood routine tests are summarized in Table 1.

**Table 1:** A breif summery of blood routine tests

Tests	11 April	15 April	16 April	21 April	29 April	Reference
	Result					
WBC (*10^3^/µL)	18.1	14.1	8.6	9.2	5.4	4.8–10.8
NE (%)	68	93.3	90.5	85.5	65.3	40–80
LY (%)	12	4.9	7.1	12.1	22.2	20–40
Hb (g/dL)	14.7	10.7	9.4	10.9	10	13.5–18
MCV (fL)	92.3	90.1	91.7	83.9	89.5	81–98
Plt (* 10^9^/L)	213	93	78	127	171	150–450
PT (s)	19.2	N.A	17.2	20.1	N.A	11–14
aPTT (s)	28	N.A	29	29	N.A	27–35
Cr (mg/dL)	1.3	1	0.8	0.7	17	07–1.4
BUN (mg/dL)	21	21	21	18	1	6–23

*WBC* white blood cell, *NE* neutrophil, *LY* lymphocye, *Hb* hemoglobulin, *MCV* mean corpuscular volume, *fL* femotoliter, *Plt* platelet, *PT* prothrombin time, *aPTT* activated partial thromboplastin time, *Cr* creatinine, *BUN* blood urea nitrogen level, *NA* not available

Electrocardiography showed sinus tachycardia, and other findings were normal. Urine analysis was positive for marijuana, benzodiazepine, and morphine. Venous Blood gas analysis was as follows: pH 6.97, partial pressure of carbon dioxide (PCO_2_) = 103 mmHg, and venous bicarbonate level (HCO_3_) = 23 mmol/L. We started the initial treatment immediately with dexamethasone (4 mg, IV every 8 h), Piperacillin/tazobactam (3.375 g, IV every 6 h), prophylactic heparin (5000 IU every 12 h). Due to significant patient-ventilator asynchrony, the patient was paralyzed with cisatracurium in addition to midazolam and propofol. During the mechanical ventilation course, he developed progressive thrombocytopenia (Table 1), but no apparent cause was found. Therefore, intravenous immunoglobulin (25 g per day for 3 days) was prescribed empirically for presumed SARS-COV2-induced thrombocytopenic purpura as idiopathic thrombocytopenic purpura. His respiratory condition got better during the next 5 days when we started weaning the patient off the mechanical ventilation, and he was extubated on 16 April 2020, but thrombocytopenia persisted.

On 21 April 2020 (10 days after admission), the patient developed upper GI bleeding associated with generalized abdominal pain with distension. Upper GI endoscopy was normal, except for the presence of minimal erythema and congestion in the cardia and fundus. No esophageal and fundal varices and duodenal ulcers were reported. Abdominopelvic Sonography showed mild free fluid in the abdominal cavity. The diagnostic abdominal tap was done, and the results are summarized in Tables 1 and 2.

**Table 2:** f.Alb, Albumin of ascitic fluid

Tests	21 April	29 April	References
	Results		
f.Alb (g/dL)	1.6	N.A	
f.pro (g/dL)	2.7	N.A	
AST (U/L)	44	40	2–37
ALT (U/L)	67	45	2–41
Alk.p (U/L)	138	138	64–306
Amylase (U/L)	42	42	20–100
Lipase (U/L)	15	17	5–60
T.B (mg/dL)	1.41	2.44	0.1–1.2
D.B (mg/dL)	0.27	0.98	0–0.3
Serum Alb (g/dL)	N.A	2.8	3.5–5.3
Serum protein (g/dL)	N.A	4.6	6.6–8.6
TIBC (µg/dL)	277	N.A	230–440
sFe (µg/dL)	49	N.A	40–168

*f.pro* protein of ascitic fluid, *AST* aspartate transaminase, *ALT* alanine transaminase, *Alk.p* alkaline phosphatase, *T.B* total bilirubin, *D.B* direct bilirubin, *TIBC* total iron-binding capacity, *sFe* serum iron, *NA* not available

**Fig. 2:**
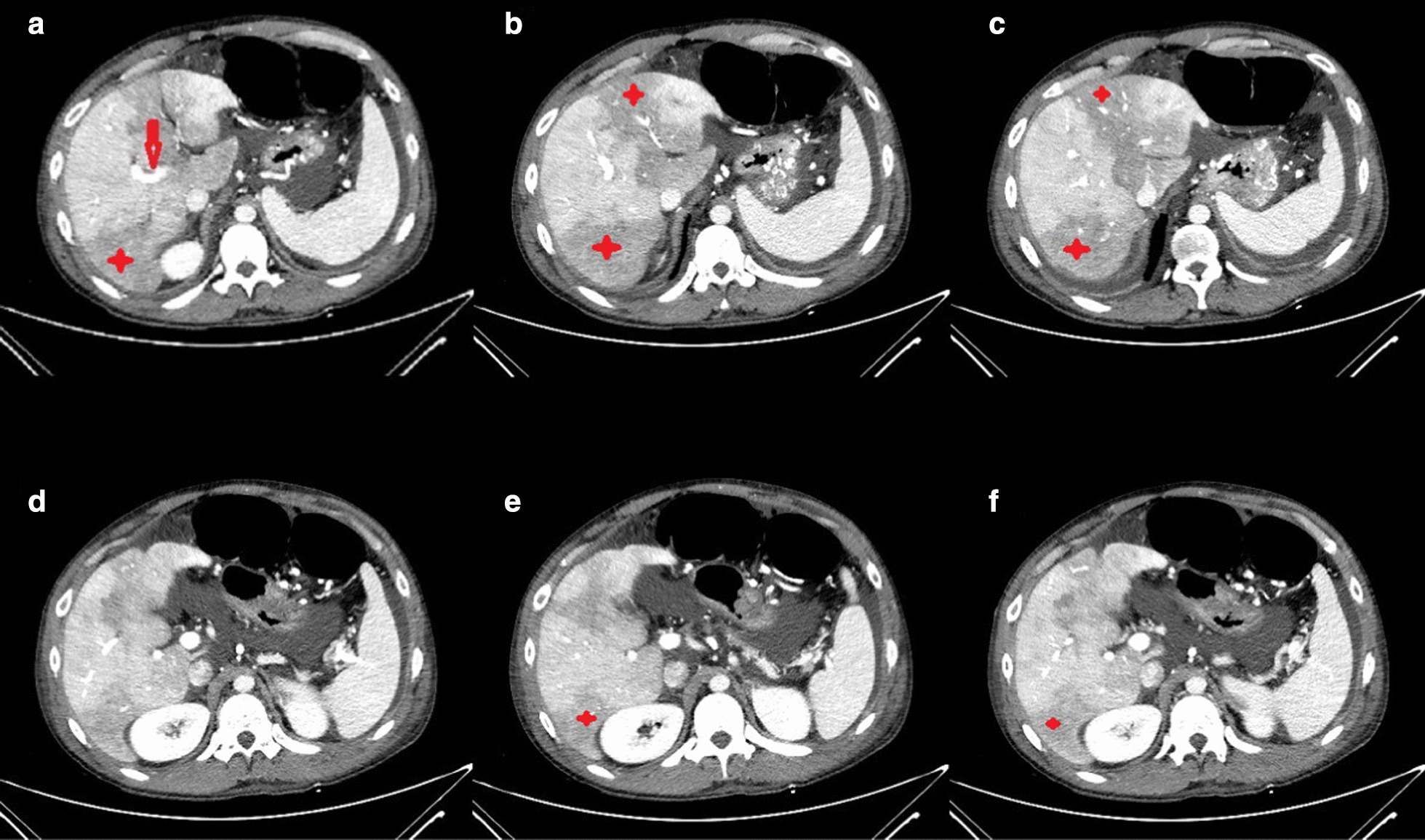
Abdominopelvic CT with IV contast. Hypoperfusion is seen in the posterior segment of right (Rt.) hepatic lobe, and the medial and lateral segments of left (Lt.) lobe (stars). Evidence of filling defect in main Rt. portal vein is also detected (arrow)

Regarding concomitant abdominal pain and abnormal liver biochemistries (Table 2), abdominal CT was requested which showed hypoperfused areas in the posterior segment of the right (Rt.) hepatic lobe and the medial and lateral segments of left (Lt.) lobe along with evidence of filling defect in the main Rt. Portal vein. Also, the Lt. portal vein was not opacified (Fig. 2). Color Doppler Sonography (CDS) of the abdomen showed the absence of flow in the portal vein without collateral veins consistent with acute portal vein thrombosis. An extensive evaluation of the possible underlying hypercoagulable state was unremarkable (Table 3).

**Table 3:** Laboratory findings in the patient

Tests	Result	Reference
C_3_ (mg/dL)	130	Adults: 90–180
C_4_ (mg/dL)	29	100–40
LAC (s)	40.8	28–42
Protein C (%)	81	65–160
Protein S (%)	89.8	65–150
Factor V Leiden (s)	91.6	≥ 120
Anti-thrombin (%)	123	70–140
ASMA	N	Up to 1:10
ANA (U/mL)	7	Negative: < 12
		Borderline: 12–18
		Positive: > 18
Anti-ds-DNA (U/mL)	1.3	Negative: < 12
		Borderline: 12–18
		Positive: > 18
β_2_ glyco-IgG (U/mL)	52.8	Negative: < 12
		Equivocal: 12–18
		Positive: > 18
β_2_ glyco-IgM (U/mL)	2.4	Negative: < 12
		Equivocal: 12–18
		Positive: > 18

*LAC* lupus anticoagulant, *ASMA* anti-smooth muscle antibody, *N* negative, *ANA* antinuclear antibody, *β*_*2*_* glyco-IgG* beta-2 glycoprotein immunoglobulin G antibody, *β*_*2*_* glyco-IgM* beta-2 glycoprotein immunoglobulin M antibody

When gastrointestinal bleeding was stopped, Enoxaparin was started at a minimum effective dose of 1.5 IU/kg/day (60 IU every 12 h) regarding recent thrombocytopenia and risk of gastrointestinal re-bleeding. After about 1 week, his abdominal pain well improved, and platelet count became normal, and finally the patient was discharged with oral warfarin. Unfortunately, patient did not come to our OPD clinic for follow up and further investigation.

## Discussion

Herein, we report a coincidence of the acute portal vein thrombosis and COVID-19 respiratory pneumonia in the background of an acute asthma attack. PVT is a rare venous thromboembolic disease typically occurring in patients with an underlying disease such as decompensated cirrhosis and/or malignancies, pancreatitis, systemic lupus erythematosus, and other hypercoagulable states [2]. Our patient did not have any of the mentioned risk factors. There is some unproven concern about the increased thrombotic event during or early after IV immunoglobulin exists. Still, no episode of portal vein thrombosis is linked to IVIG to date [3]. Several studies showed that thromboembolic events increased in patients with SARS-CoV-2 infection since the pandemic declared by the World Health Organization (WHO) [4–6]. In previous studies, thromboembolic events were pulmonary emboli, deep venous thrombosis (DVT), and cerebral infarction usually occurring in the elderly with severe COVID-19 and comorbid diseases such as hypertension and diabetes [5–8]. To the best of our knowledge, our patient is the third case with PVT. The first was a 79-year old female without unknown medical comorbidities who had diarrhea and epigastric abdominal pain and acute dyspnea 8 days after abdominal pain [9]. The second case was a 72-year-old male with Parkinson’s disease and mild vascular dementia who was suffering from jaundice, fever, hypotension, elevated liver enzyme, and acute kidney injury. At first, he was admitted with the impression of Escherichia Coli sepsis. Therefore, he received proper antibiotic regimen, but the fever was not resolved [10]. However, in our study, the patient was a young man without any significant comorbidities, except for a history of asthma and chronic alcohol consumption.

The exact pathophysiology of the increased prevalence of thromboembolism events in COVID-19 positive patients remains unclear [5]. However, immobilization and hypoxemia, as seen in our patient at the time of admission along with the increase in inflammatory cytokines such as interleukin-6 (IL-6), IL-10, and Tumor Necrosis Factor Alfa (TNF-α) are the possible mechanisms [11–13]. Unfortunately, we cannot assess the serum level of these cytokines. The attachment of the viruses to the endothelial surfaces via angiotensin-converting enzyme receptor (ACE-R), leads to the lymphocytic endothilitis, interferon-1 (INF-1), and prothrombotic genes overexpression [14]. *Becker* speculated that the attachment of COVID-19-related extracellular RNA to the factor XI and XII which belongs to the contact pathway of the coagulation cascade leads to the interferon-γ (INF-γ) suppression and a hyper-inflammatory state [15]. Our patient was a smoker. Smoking has been deemed as a risk factor for severe COVID pneumonia and a minor risk factor for thrombotic event which may potentiate the above-mentioned hypercoagulable state [16].

## Conclusion

Our report of portal vein thrombosis in the setting of COVID-19 pneumonia adds portal vein thrombosis to other COVID-19 associated thrombotic phenomenon and denotes that the clinician who cares for these patients should have a high index of suspicion to diagnose this unusual presentation in a proper clinical scenario.

## Data Availability

The datasets generated and/or gathered during this study are not publicly available given our commitment to patient privacy rights. However, anonymous data may be requested from the corresponding author for valid use.

## Abbreviations

PVT: Portal vein thrombosis
ED: Emergency department
N/V: Nausea and vomiting
CT: Computed tomography
CXR: Chest X-ray
RT-PCR: Real-time polymerase chain reaction
Rt.: Right
Lt.: Left
CDS: Color doppler sonography
WHO: World health organization
DVT: Deep venous thrombosis
IL: Interleukin
ACE-R: Angiotensin-converting enzyme receptor
INF: Interferon
